# Sharing Research Data and Intellectual Property Law: A Primer

**DOI:** 10.1371/journal.pbio.1002235

**Published:** 2015-08-27

**Authors:** Michael W. Carroll

**Affiliations:** Program on Information Justice and Intellectual Property, American University, Washington College of Law, Washington, D.C., United States of America

## Abstract

Sharing research data by depositing it in connection with a published article or otherwise making data publicly available sometimes raises intellectual property questions in the minds of depositing researchers, their employers, their funders, and other researchers who seek to reuse research data. In this context or in the drafting of data management plans, common questions are (1) what are the legal rights in data; (2) who has these rights; and (3) how does one with these rights use them to share data in a way that permits or encourages productive downstream uses? Leaving to the side privacy and national security laws that regulate sharing certain types of data, this Perspective explains how to work through the general intellectual property and contractual issues for all research data.

## Abstract 2

This Perspective addresses questions and concerns that biologists often have about intellectual property rights in research data and how these interact with the deposition or sharing of data.

For the researcher seeking to use another’s data, this Perspective offers some good news and some not as good news. The good news is that if a source of data—the researcher or repository—gives permission to reuse the data and one’s intended use fits within the scope of the permission, one need not be overly concerned with the details of the discussion that follows because the permission provides the legal basis for data reuse. For example, if one seeks data from the European Bioinformatics Institute, one will find that the terms of use state that “[t]he public databases of EMBL-EBI [The European Molecular Biology Laboratory-The European Bioinformatics Institute] are freely available by any individual and for any purpose” [1]. This would appear to give any individual academic researcher permission to copy and reuse the data at will. It leaves open a question about whether an employee acting on behalf of his or her employer (is s/he acting as “an individual”?) is equally granted this permission.

There is, however, a catch. The EBI’s terms also warn the user that some third parties may claim intellectual property or other legal rights on the original data, and it is up to the researcher not to infringe these rights. This kind of legal uncertainty interferes with the productive reuse of research data. It can be avoided if the repository requires depositors to grant permission to downstream users or to give up any intellectual property rights they may have in the data. Alternatively, the final section of this Perspective describes means by which repositories can make it easy for depositors to signal the scope of the permission they grant to downstream users.

In the absence of clear permission, mapping how intellectual property law does—and does not—apply to research data may be of use. In my view, the law makes all of this far more complicated than it need be. For those seeking to pick and choose which reuses of another’s data may be permitted by law, regrettably, the answers to the above questions are more context dependent than many would like.

This is so for two reasons. First, the source of all intellectual property rights is national law. Certain international treaties harmonize intellectual property owners’ rights but leave the users’ rights to vary by country. Second, certain countries have added protection beyond what the treaties require. Specifically, the members of the European Union, candidate countries in Eastern Europe, Mexico [2], and South Korea have created a specialized database right that applies to certain databases created or maintained within their borders. These laws regulate uses of these databases only within their borders.

## What Are the Legal Rights in Data?

The rights that may apply to research data are trade secrets (confidential information), copyrights, and special database rights in the EU and South Korea. Patents may apply to some forms of data, but the more common issue is that data sharing may have implications for the acquisition of patent protection in inventions that arise from research. Finally, the ability to use contracts overlays all of these rights and can be used to provide permission for reuse through licensing of underlying rights (but also to restrict reuse merely as a term and condition of granting access to data). Focusing on the case of a researcher depositing data in compliance with a journal’s publication policies, the following discusses the relevant rights and their application.

### Trade Secret (Also Known As Proprietary or Confidential Information)

Most scientific researchers own trade secrets in their research data for some period of time, even if they are unaware of this fact. This is because, according to international standards, national laws treat information as a trade secret if it derives economic value from not being generally known or readily ascertainable, so long as the information has been subject to reasonable measures to keep it secret. Most research data meets this definition, at least in the early stages of collection or generation.

The ease with which trade secret protection is acquired is mirrored by the ease with which it is lost. Public disclosure of the information removes any associated trade secret protection because the information has become generally known or readily ascertainable. In commercial practice, trade secrets are routinely created and destroyed as companies develop new products and services in confidence that they then publicly disclose when they go to market. Analogously, trade secrets in research data are routinely removed through data sharing practices, including depositing in a publicly accessible repository.

In traditional academic research, trade secrecy is unlikely to be invoked unless a member of a research team decamps to another team with confidential data. The issue becomes more salient in the context of industrial research or commercially sponsored academic research. Most commercial sponsors provide for the management of trade secrets in the terms of their sponsorship agreements [3]. For example, if a researcher collaborates with a pharmaceutical company, the researcher may be contractually bound to suppress the release of research data until the sponsor has developed a patentable product. Academic researchers and their offices of sponsored projects should carefully review drafts of sponsored research agreements and clinical trial agreements to ensure they do not inappropriately restrict a researcher’s right to disseminate the results of the scientific research they have conducted. A researcher should ensure that the agreements do not permit commercial sponsors to revise, delete, or suppress information generated by the researcher. The terms and timing of disclosing research results that are trade secrets should be incorporated into the sponsored research agreements, not negotiated at the time of publication [3,4].

### Copyright

Copyright grants the author(s) of an original work the exclusive rights to reproduce the work, to publicly distribute copies, to publicly display, publicly perform, or otherwise communicate the work to the public, and to make adaptations of the work.

Understanding how copyright applies to the sharing of research data is more work than it is worth unless it is likely or plausible that the creator, owner, or repository in which data resides is likely to seek to limit copying, distribution, or other reuses of data. When such rights of control are likely to be asserted or when a third party requires evidence that all permissions for republication or reuse of data have been obtained, copyright law plays a limited but inescapable role in the sharing of research data.

Copyright law is founded on certain science-friendly policies. Copyright imposes no restrictions on the sharing of the basic building blocks of knowledge—facts and ideas—which are part of the public domain. Researchers routinely rely on this freedom to copy in their daily practice [5]. For example, the freedom to copy ideas has been an important component of the rapid propagation of the CRISPR/Cas9 (Clustered Regularly Interspaced Short Palindromic Repeats/CRISPR associated protein 9) process for gene editing [6]. (There is a pending patent dispute about applications of this method [7], but the underlying idea that one can manipulate bacterial immune response to splice genes is in the public domain.) Similarly, raw observational and experimental data are “facts” for copyright purposes that are free to be shared and reused without copyright restriction [5].

Copyright applies to original works of authorship. For copyright purposes, an author is one who makes creative or editorial decisions about how ideas and facts are expressed. For example, the only authors of a journal article for copyright purposes are those who wrote the words or created the tables or figures. The amount of creativity or editorial discretion needed to create a work of authorship is quite minimal. As a result, some aspects of a dataset are likely to have a copyright attached to them. Copying the whole dataset will involve copying the copyrighted layer. Additionally, separate copyrights can attach to data items, organizational structures, and metadata (Box 1).

**Box 1. Layers of Copyrights in Databases**

Copyright at the item level is limited to items that involve expressive choice, such as drawings or photographs. For example, if one treats the images in the Encyclopedia of Life as data items, the very large majority of these have enough creative expression to be copyrightable. However, the copyright is limited to the expression that the author created. One would not be exercising any rights under copyright by creating a drawing of an animal depicted in a photograph. The photographer is not the author of the animal’s characteristics. The author’s copyright is limited to this particular expression through the way the shot is composed, lit, and focused, for example. Otherwise, at the item level, most data expressed as numeric values are likely to be “facts” that are in the public domain. This means that even if there is a copyright at the organizational level, these numeric values can be copied and reused without any copyright restrictions.

At the organization layer, a separate copyright can arise with respect to the manner in which data are selected and arranged. For example, even the organization of an Excel spreadsheet could be copyrightable if a researcher exercised discretion in selecting field names and arranging their order. However, the copyright that would arise would be limited to this layer of the dataset. Another researcher would not be infringing any of the rights associated with this work if s/he were to republish the data in a spreadsheet with renamed and reorganized fields. As the amount of organizational choice increases in, for example, the structure of a relational database, the amount of copyrightable expression increases as well.

Annotations, visualizations, and other forms of metadata can receive separate copyright protection if they are sufficiently original. Creating visualizations, figures, charts, graphs, and other forms of “processing” of research data often involves the kinds of discretionary decisions about expression to which copyright applies, and copyright becomes an issue for a user who seeks to reuse these forms of original expression. Finally, compilations of datasets—used in meta-analysis, for example—might receive a separate copyright if the selection and arrangement of these involve sufficient discretionary choice. Such a copyright would apply only to this selection and arrangement and not to any of the underlying items or organizational features of individual datasets.

In cases in which copyright attaches to some aspect of research data relevant to a potential user, it becomes important to know which copyright(s) regulate(s) a proposed use. These rights in the copyrighted layer of a dataset give the owner a legal hook to seek to control the reproduction or distribution of datasets and visualizations.

When copyright does govern a proposed use of data, the use may be permitted by users’ rights that are expressed as exceptions or limitations to the copyright owner’s rights. These users’ rights vary by country or region (Box 2). For example, countries whose law is based on that of the United Kingdom have a flexible provision called fair dealing that resembles fair use but is somewhat more limited. A fair dealing analysis involves a first step of determining whether the use fits within one of the categorically eligible types of use. Using a copyrighted work for noncommercial research or private study or criticism or review are examples of categorically acceptable uses. Such a use does not infringe copyright if it is “fair dealing,” which is determined by balancing similar considerations about the purpose of the use, the extent of the work used, and the effect of the use on the copyright owner. In the rest of Europe, countries also have the option—but not a requirement—to provide exceptions for these same uses. The picture of users’ rights becomes even more of a patchwork as one extends the lens to the rest of the world.

Countries also provide authors with some level of moral rights in their works of authorship. These rights are personal to the author and cannot be transferred. Authors have the right to be attributed as such. Authors also have the right to not be attributed if they no longer wish to be associated with the work. A strong version of moral rights even gives the author the right to retract a work from publication and to enjoin any further publication or duplication. Other rights include the right of integrity in the work, which limits adaptations to those that do not harm the reputation of the author. Of these, the attribution right is likely the one with the most salience in the context of data reuse.

**Box 2. National Variation in Users’ Rights in Copyright Law**

The scope of copyright control is limited by statutory limitations and exceptions to the copyright owner’s exclusive rights that permit certain reuses by law. These limitations and exceptions have not been harmonized internationally. As a result, the freedom to use the copyrighted layer of a dataset—by, for example, copying the whole set—without permission depends upon the country in which the copying takes place. This is a prime example of how and where the law is far more complex than necessary to chart the basic rules for when data sharing is permitted by law and when the presence of a copyrighted layer would require the copyright owner’s permission.

All countries have a targeted list of uses that are permitted by law, but these lists vary considerably, and the identified uses can often be defined quite specifically and narrowly. For example, the UK recently amended its copyright law to explicitly permit researchers to content mine the research literature because its Parliament was uncertain whether the existing limitations and exceptions would permit the copying necessary to engage in content mining [8].

A number of other countries also have a flexible exception that requires a balancing of considerations to determine whether the use is permitted. The most clear-cut example is the fair use doctrine in the United States and Israel. Under this rule, one considers the nature and purpose of the use, how much authorship is in the source work, how much of the author’s expression has been taken in the use, and whether the use has an adverse effect on the copyright owner’s ability to economically exploit the work. Relevant to this discussion, courts have found that copying the copyrighted layer of a work is fair use if the purpose is to extract the public domain information incorporated in the work.

### Sui Generis Database Rights—Europe and South Korea

In the EU, certain candidate countries in Eastern Europe, and South Korea, research data may also be subject to a special database right. Mexico also protects databases that do not qualify for copyright protection, but its measures are not discussed here. Keep in mind that what follows applies only to (1) databases that are created or maintained within the borders of EU member states or South Korea and (2) uses of these protected databases that take place within these territories. As frustrating as this may be to a globalized research community, in a narrow class of cases, this right could apply to a download of a substantial amount of data that takes place on a computer connected to the Internet in Europe or South Korea, but not elsewhere.

Under the EU’s Database Directive [9], these special rights apply to any database that requires a “substantial investment” to assemble or maintain. As interpreted by the Court of Justice of the European Union (“Court of Justice”), this right is limited to those databases that require investments in the obtaining of data, not the creation of the underlying data [10]. This means that a sole source database, like a sporting events schedule, generally does not enjoy protection, while publishers of directories or lists can maintain protection if they only obtain data from others, not create it themselves. This sui generis right in the nonoriginal (i.e., not subject to copyright) portions of a database lasts for 15 years.

Sui generis database rights protect against the extraction or reutilization of substantial parts of a protected database as well as frequent extraction of insubstantial parts of a protected database. This legal right would be a significant barrier to sharing research data were it not subject to a limitation for noncommercial research. A great deal of research data likely meets the threshold requirement of “substantial investment” of financial resources and labor because of its capacious definition, but a substantial amount of university or nonprofit hospital use of such data likely qualifies for the limitation. A risk remains that increased commercial sponsorship of academic research may test the boundaries of this “noncommercial” exception.

One user-friendly provision of the Database Directive is that it greatly limits the ability of a database owner to use terms of use or other forms of contractual agreement to add use restrictions that exceed those in the Directive—by, for example, prohibiting the occasional extraction or republication of insubstantial amounts of data taken from the database. In a recent odd twist, the Court of Justice has determined that if a database lacks both copyright protection and protection under the Directive, then the owner’s terms of use will be enforceable [11].

### Patent

The impact of disclosing or sharing research data on patent rights can be easily overstated by those seeking legal cover to avoid sharing data. However, the issue is not entirely fabricated because there are situations in which data sharing may have an adverse effect on a party seeking patent protection.

Patents are exclusive rights in inventions. An invention is patentable if it is new, useful, and demonstrates an inventive step over what is already known within the relevant field of knowledge. Unlike the rights described above, patents only arise if they are applied for and granted by a public authority. In most countries, the application process requires an examination to determine if the legal requirements for patent protection are met. In a few countries, such as South Africa, one need only register one’s claim to receive a patent. As with other intellectual property rights, a patent applies only to uses that take place at least in part within the borders of the country from which a patent has issued.

The putative risk of data sharing arises because public disclosure of an invention prior to filing a patent application can destroy or impair one’s right to obtain patent protection for the invention [3]. However, most research data are not eligible to be protected as inventions as such. (Whether research data is capable of being a patentable invention depends upon how elastic one’s definition of “data” is. If genetically modified organisms are “data,” for example, then such data very likely are eligible for patent protection and any intended patent applications should be filed prior to their public disclosure.) Instead, the invention is far more likely to be disclosed through the publication of an associated research article than by the sharing of data.

When a published research article teaches the public everything about inventions arising from research that data deposit does, then the deposition has no more impact on patentability than the decision to publish had. For this reason, the rules that researchers must abide by for disclosing inventions to their university or other employer or funder prior to publishing a research article should be read to include disclosure prior to depositing associated data as well [3,12].

There may be cases in which data deposit has a marginal additional impact on patentability of inventions arising from research reported in an article. One such case would be when the article does not describe the invention but the data do. Another case would be one in which the data disclosure fills a gap in other researchers’ knowledge such that inventions that arise from the research are not described by the data but rendered “obvious” to one skilled in the art by the disclosure. Once an invention becomes obvious, it lacks the required inventive step needed to obtain protection.

A separate patent issue for data sharing arises when a patented process claims the steps involved in data sharing or reuse. A patent grants the owner the rights to exclude others from making, using, selling, offering to sell, or importing the invention. Use of an invention is interpreted quite broadly. A patentable process could claim a series of steps that would be practiced in connection with certain forms of data reuse. This issue is so context dependent that little more than raising it as a consideration can be done here.

## Who Holds These Rights?

This question becomes relevant when one wishes to assert intellectual property control or when one must seek permission to make an intended use of another’s research data. Usually the person who creates or generates the intellectual property is the initial owner of these rights. When the creator is an employee, determining who holds the rights becomes more complicated, and national variation reemerges as an issue. Finally, all of these rights are transferable (except moral rights in copyright), so the initial owner may no longer be the rights holder.

### Trade Secret

Employers generally own trade secrets that are developed by their employees within the scope of their employment. This rule certainly encompasses the research data generated or collected by an industrial researcher. Whether or how this rule applies in the academic research context is not clear. In the absence of an agreement or policy that applies to trade secrets, student or independent researchers would own any trade secret rights associated with their data. Whether an employee of a university or hospital creates or collects data within the scope of employment is a subject of theoretical interest. In practice, however, the rules of ownership are routinely altered or determined contractually. Sponsored research agreements and university or hospital intellectual property policies generally establish the rules for ownership and disclosure of trade secrets [3,4].

### Copyright

Copyright is owned initially by the author(s) of a copyrighted work. For copyright purposes, the author is the person or persons who make the creative or editorial decisions about how to express the underlying facts and ideas. This is a much more constrained version of authorship than applies in science. This gap between what science and copyright law values is readily seen in how credit is distributed for a scientific publication. Scientists recognize that results emerge from team effort, and scientists have developed conventions about who is listed as an author and in what order to signal this recognition to the broader community. For copyright purposes, however, only those members of the team who expressed themselves by writing the words, drawing the figures, or otherwise creating original expression are authors with rights under copyright.

Thus, if there is a copyright layer to a dataset or database, the owner(s) of the copyright(s) associated with this layer would be the one(s) who chose how to organize, arrange, annotate, or visualize the data rather than the one(s) involved in its generation or collection [5].

When the copyrighted work is created by an employee within the scope of employment, a national division emerges. In the US, under the work-made-for-hire rule, the employer is treated as the author, and the employee has no rights [13]. Whether this rule applies to the research and teaching materials created by university employees is the subject of a division of opinion. Some argue the rule does not apply to research outputs either because the particular research from which the data arise may not be considered within the scope of employment or because prior law had recognized a “teacher exception” to the rule that may have been implicitly carried forward into current law. On its face, current law does not state any exceptions to the rule. In the rest of the world, the individual creator(s) start(s) with the rights, but these may automatically be transferred to the employer if the employment agreement provides for this.

### Sui Generis Database Rights—Europe and South Korea

The holder of sui generis database rights is the person or entity that makes the substantial investment in collecting data from other sources or maintaining the database. In the research context, these rights usually will belong to data aggregators and repositories rather than individual researchers or research teams.

### Patent

Patent applications generally must be filed in the name of the inventor(s). The rights in the patent, however, can be assigned to another party. By agreement, employees routinely assign the rights in their inventions to their employer. University and hospital employment agreements and policies often require that researchers assign rights to inventions arising under sponsored research agreements to their employer as well. Academic institutions sometimes hold more patents than both the government and commercial businesses. For example, the University of California and The John Hopkins University were both in the top 15 holders of deoxyribonucleic acid patents in 2004 [14].

## Recommendations for Increasing Data Sharing and Openness

### Contracts and Licenses

When one or more intellectual property rights apply to research data, the owner of such rights can grant permission for reuse through a license. In legal terms, a grant of permission is a nonexclusive license. An exclusive license is one in which the rights holder agrees to give up any rights to use the intellectual property, usually in return for some form of compensation.

From a legal perspective, terms of use or other “licenses” fall into one of two groups. In the first group, there is an underlying intellectual property right associated with data that would be violated by the user in the absence of the permission granted by the terms. That is an intellectual property license. Violation of such a license could lead to a court order requiring the user to cease any further use. Damages and attorneys’ fees may also be assessed against the breaching user.

In the second group, there is a collection of data that has no underlying intellectual property right associated with it, such as a large collection of sensor data that is organized in an unoriginal manner—say, chronologically. If one were to download these data from a site with “terms of use” associated, those terms are still enforceable as a contractual agreement, but there would be no intellectual property right to infringe. Enforcing any use restrictions in this second group of agreements is much more difficult because the author of the terms has to prove that the use has caused measureable economic damages.

Although there are policy arguments against enforcing the terms of use in this second group—because they impose use restrictions on data that intellectual property law treats as in the public domain—courts in the US and elsewhere generally have found these terms of use to be enforceable as long as the basic requirements for voluntary agreements have been met. For example, a Maryland district court upheld a terms-of-use agreement even when a third-party user obtained database access merely by clicking a box to accept, but failed to review, the terms of use [15].

Since the practice is legal and enforceable, it should be a topic for community discussion whether it is ethical or appropriate to condition access to data on agreement to a contract that imposes use restrictions on data that is otherwise free of any intellectual property rights.

### Clarifying the Terms of Use

As the discussion above demonstrates, it is not often clear to a potential user of data whether any intellectual property rights are associated with the data and, if there are, who owns these. To promote data reuse, it is incumbent on the owner(s) of these rights to mark the data with the associated permissions. Otherwise, one ends up with the muddy rules set forth by the EBI outlined at the opening of this Perspective.

### Removing or Limiting Rights Restrictions

#### Trade secret

The easiest way to grant permission to use a trade secret associated with data is to get rid of it by publicly disclosing or depositing the data. Otherwise, some form of confidentiality or nondisclosure agreement would be needed to preserve the trade secret(s) while permitting their reuse by a closed group of other researchers.

#### Patent

As discussed above, public disclosure can also limit or destroy the ability to obtain patent rights in inventions associated with data. When a patent covers the collection, generation, or use of research data, the owner can grant permission to practice the process through a nonexclusive license or through a public statement that the patent will not be asserted against researchers practicing the process in connection with their research.

#### Copyright and database rights

These rights are more persistent than trade secrets or patents. However, they also can be permanently removed in most parts of the world if the owner of the rights publicly and unequivocally states his or her intention to permanently relinquish these rights. Creative Commons provides a tool called CC0 (CC Zero) to accomplish this task. In countries that deny owners the right to relinquish these rights (yes, it happens), CC0 functions as a license that imposes no constraints on the user (Box 3).

**Box 3. Creative Commons**

Creative Commons is a global organization that promotes the sharing and reuse of creative, educational, and scientific works by supplying standardized public licenses that anyone can use to permit reuse of works they created or to which they own the rights. The primary tools are six copyright licenses, a copyright waiver, and a label that indicates that a work is free from copyright and in the public domain. The six licenses and the CC0 waiver are designed to respond to creators who have different appetites for reuse of their works. As is indicated in the body of this Perspective, CC0 is a way to dedicate a work to the public domain by waiving all rights under copyright and any sui generis database rights that may apply. This tool is used by those who create public domain clipart, for example, and in connection with sharing data for which copyright is only an incidental consideration. Unlike CC0, the licenses impose some conditions on reuse.

**The licenses**

The broadest license is the Creative Commons Attribution (CC BY) license, which requires only that the user provide attribution as directed by the licensor. This license is used by open access publishers, including PLOS, by creators of open educational resources, such as OpenStax College and Rice Connexions, and by a range of other creators. All of the other licenses keep the attribution requirement and add other conditions. One of these is the “Share Alike” requirement, which provides that anyone who adapts the licensed work must license the adaptation under the same license as the source work. This requirement is a close cousin to certain “copyleft” licenses used for software, such as the GNU General Public License (GNU’s Not Unix!). Wikipedia uses this Creative Commons Attribution-ShareAlike (CC BY-SA) license, and only materials licensed under CC BY or CC BY-SA can be uploaded to Wikimedia Commons.

The Creative Commons Attribution-NonCommercial (CC BY-NC) license limits licensed uses to noncommercial uses. Last, one may permit only copy-paste reuse and not license the creation of derivative works by using the Creative Commons Attribution-NoDerivs license (CC BY-ND). The final two licenses combine the noncommercial condition with either the Share Alike or the No Derivatives condition (https://creativecommons.org/licenses/). This may seem like more complexity than it is worth, and some critics of Creative Commons take this position, but a quick look at the uses of these licenses on Flickr demonstrates that creators appear to want this full choice set to share their works (https://www.flickr.com/creativecommons/).

Alternatively, one can grant the public permission to use copyrights associated with a dataset or database through a license. For example, a researcher may post to the web a complex dataset that has an original database model. Users who copy the dataset merely to extract the uncopyrightable data elements would not need permission to do so in much of the world. However, if one were to republish the full dataset, one would be using the copyright layer in a manner that likely would require a license. The researcher publishing the dataset may simply want to require that any republication be done with proper attribution. The researcher could write a bespoke license to require this or could use a standard copyright license, such as the CC BY license. The organization publishes an FAQ on the relation of its licenses to databases on its website [16].

## Abbreviations

CC: Creative Commons
CC0: Creative Commons Zero
CC BY: Creative Commons Attribution
CC BY-SA: Creative Commons Attribution-ShareAlike
CC BY-NC: Creative Commons Attribution-NonCommercial
CC BY-ND: Creative Commons Attribution-NoDerivs
CRISPR/Cas9: Clustered Regularly Interspaced Short Palindromic Repeats/CRISPR associated protein 9
EMBL-EBI: The European Molecular Biology Laboratory-The European Bioinformatics Institute
GNU: GNU’s Not Unix!
